# THE USE OF MINDFULNESS-BASED INTERVENTION FOR LONG-TERM CARE RESIDENTS: A REVIEW OF THE LITERATURE

**DOI:** 10.1093/geroni/igae098.3174

**Published:** 2024-12-31

**Authors:** Audrieanna Raciti, Yu-Ping Chang

**Affiliations:** Buffalo, New York Mills, New York, United States; University at Buffalo, SUNY, Buffalo, New York, United States

## Abstract

Physiological and psychological health is a significant concern among people residing in long term care. Researchers have begun to design and test mindfulness-based interventions (MBIs) as a strategy to lessen the burden of these impacts on long-term care residents as well as our already strained healthcare system. The purpose of this review is to synthesize the use of MBIs and their impact on health-related outcomes and quality of life among long term care residents. Six databases: CINAHL, PUBMED, Cochrane, Web of Science, Psychinfo, and Embase were searched to identify original studies leveraging the use of mindfulness to improve health-related outcomes for long term care residents. Multiple keywords were used including mindfulness-based stress reduction, meditation, mindfulness intervention, long-term care, nursing home, residential care, older adult, elderly, and aged. Nine studies meeting the selection criteria were included in this systematic review. Most of the studies have a small to medium sample size. The types of MBIs include mindfulness-based cognitive therapy, silent meditations, mountain meditations, loving-kindness meditations, awareness exercises, breathing exercises, visualization, and guided imagery. The majority of those MBIs demonstrate a significant improvement in alleviating symptoms of stress, depression, anxiety, relocation anxiety, worsening dementia, and other mental health concerns, while one study also showed significant improvement in A1C levels among residents with type 2 diabetes. However, some studies have a high attrition rate. Future research is needed to utilize a more rigorous design with a large sample size and longitudinal studies. Participant engagement should be considered as part of the intervention activities.

